# Human β-defensin-2 production from *S. cerevisiae* using the repressible *MET17* promoter

**DOI:** 10.1186/s12934-017-0627-7

**Published:** 2017-01-18

**Authors:** Thea S. B. Møller, Joanna Hay, Malcolm J. Saxton, Karen Bunting, Evamaria I. Petersen, Søren Kjærulff, Christopher J. A. Finnis

**Affiliations:** 1 Novozymes Biopharma UK Limited, Castle Court, 59 Castle Boulevard, Nottingham, NG7 1FD UK; 2 Department of Physics and Nanotechnology, Aalborg University, Skjernvej 4A, Aalborg East, 9220 Aalborg, Denmark

**Keywords:** *MET17* promoter, *Saccharomyces cerevisiae*, Yeast, Human β-defensin-2

## Abstract

**Background:**

Baker’s yeast *Saccharomyces cerevisiae* is a proven host for the commercial production of recombinant biopharmaceutical proteins. For the manufacture of heterologous proteins with activities deleterious to the host it can be desirable to minimise production during the growth phase and induce production late in the exponential phase. Protein expression by regulated promoter systems offers the possibility of improving productivity in this way by separating the recombinant protein production phase from the yeast growth phase. Commonly used inducible promoters do not always offer convenient solutions for industrial scale biopharmaceutical production with engineered yeast systems.

**Results:**

Here we show improved secretion of the antimicrobial protein, human β-defensin-2, (hBD2), using the *S.* *cerevisiae MET17* promoter by repressing expression during the growth phase. In shake flask culture, a higher final concentration of human β-defensin-2 was obtained using the repressible *MET17* promoter system than when using the strong constitutive promoter from proteinase B (*PRB1*) in a yeast strain developed for high-level commercial production of recombinant proteins. Furthermore, this was achieved in under half the time using the *MET17* promoter compared to the *PRB1* promoter. Cell density, plasmid copy-number, transcript level and protein concentration in the culture supernatant were used to study the effects of different initial methionine concentrations in the culture media for the production of human β-defensin-2 secreted from *S.* *cerevisiae*.

**Conclusions:**

The repressible *S. cerevisiae MET17* promoter was more efficient than a strong constitutive promoter for the production of human β-defensin-2 from *S. cerevisiae* in small-scale culture and offers advantages for the commercial production of this and other heterologous proteins which are deleterious to the host organism. Furthermore, the *MET17* promoter activity can be modulated by methionine alone, which has a safety profile applicable to biopharmaceutical manufacturing.

## Background

*Saccharomyces cerevisiae* has a long safe history of use for the production of biopharmaceutical proteins and has a rich density of knowledge detailing its genetics, biochemistry, physiology and large-scale fermentation performance. Many different promoters have been used to successfully drive the expression of foreign genes in *S.* *cerevisiae*. However, the choice of promoter for heterologous gene expression can affect the product yield greatly [1, 2]. Expression using regulated promoter systems is advantageous for proteins which are toxic to the host cell as this allows controlled timing of gene expression and higher production levels. Regulated expression can be achieved through manipulation of the growth medium by adding metabolites. The galactose (GAL1-10) promoters are commonly used to allow regulation of the target gene by the carbon source; using galactose for induction and glucose for repression [3]. However, the commonly used galactose promoter systems are not always applicable for industrial expression systems, as the *S.* *cerevisiae* strains used may not respond well to galactose. In addition, regulation of these promoters may interfere with the cellular metabolism and in many cases the regulation is not tight enough to completely shut off transcription. This issue was addressed by use of the tetracycline (Tet-On/Off) promoters, which are either inducible or repressible [4]. Here, gene expression is activated as a result of binding of the Tet-Off or Tet-On protein to an element located within an inducible promoter. One advantage of this system is that promoter regulation with the tetracycline derivative, doxycycline does not interfere with the yeast cellular metabolism. However, doxycycline is not ideal for use in biopharmaceutical processes and its regulation needs tetracycline-regulated activators and repressors, which require a specific strain background or additional manipulations of the strains in use [4, 5]. Consequently, alternative promoter systems with safe and simple regulation are desirable for some large scale biopharmaceutical processes.

In *S. cerevisiae*, the *MET17* gene (also known as *MET15* and *MET25*) encodes *O*-acetylhomoserine sulfhydrolase [6, 7] and its promoter displays repression of transcriptional expression in the presence of methionine [8] or *S*–adenosylmethionine (SAM) [9, 10]. *MET17* catalyses the last step of the sulfate assimilation pathway in *S.* *cerevisiae,* which is the incorporation of sulfide into a carbon chain [7]. The promoter is efficiently and strongly repressed at high methionine concentrations with the expression of *O*-acetylhomoserine sulfhydrolase only occurring below 0.05 mM methionine [6, 7, 11]. Utilising media with the correct concentration of methionine, the *MET17* promoter has been used previously to express human serum albumin (HSA) and albumin fusion proteins, including repressing production of glucagon-HSA in early log phase with expression in late log phase [6]. This separation of the growth and production phases may be especially useful for expressing proteins that are toxic to the yeast host, which are best produced in the late log phase. However, a separation in growth and production phases cannot be achieved using constitutive promoters, where the secretion of toxic, or even relatively non-toxic proteins, can be deleterious to the host resulting in reduced product yields. Here, the adverse effects of the recombinant hBD2 on the host may be either intracellular during secretion, or extracellular due to its accumulation to toxic levels in the growth media. Compared to inducible systems such as the galactose system, the *MET17* promoter does not require a change in carbon source that may potentially slow growth, or the addition of an inducing metabolite [6]. This system is based upon the consumption of methionine from the media leading to subsequent expression of the gene of interest downstream of the *MET17* promoter. In the past, the *MET17* promoter has been used to produce several heterologous proteins under derepressing conditions, including β-galactosidase [12], CaArn1, a siderophore transporter from *Candida albicans* [13], green fluorescent protein (GFP) and GFP fusions [14], and human albumin and albumin fusions [6].

Here we describe the use of the repressible *MET17* promoter for the secretion of human β-defensin-2 in shake flask cultures (SFC) using a highly productive *S. cerevisiae* strain. This yeast strain was initially developed for the secretion of recombinant human albumin (rHA); however, studies have shown that it can also be used to express a diverse range of heterologous proteins [15]. hBD2 belongs to the intriguing class of antimicrobial and immunomodulatory peptides called defensins. Defensins are small cationic and cysteine-rich peptides that play a crucial role in the host defence against microorganisms [16, 17]. hBD2 is a 41 amino acid peptide first characterised in psoriatic skin, which has been shown to be active against Gram-negative (*E. coli*, *P.* *aeruginosa*) and Gram-positive (*S. aureus*) bacteria as well as yeast (*C. albicans*, *C. krusei*, *C. parapsilosis*) [18–20]. The action of defensins against microorganisms generally involves membranolytic disruption, permeability, or pore formation [21]. Their amino acid composition, amphipathicity, cationic charge and size allow them to attach to and insert into membrane bilayers to form pores [22].

Heterologous expression systems have the potential to provide hBD2 for both clinical research and therapeutic applications. However, the issues of host cell toxicity, proteolytic degradation, folding and low yields must be overcome to provide a suitable platform for commercial production. To date, recombinant hBD2 has predominantly been produced from inducible *E. coli* expression systems as a fusion protein requiring in vitro cleavage [23]. Subsequent oxidation may also be needed, for which yields of 1–2 mg final product per litre of bacterial culture have been reported [24]. However, soluble active hBD2 can also be obtained from fusion proteins without the need for refolding, where yields estimated to be around 100 mg/L purified mature hBD2 have been obtained after enterokinase cleavage [25]. In contrast, *S.* *cerevisiae* offers the advantage of secreting fully folded mature bioactive hBD2 into the culture medium at relatively high product titres without the need for costly in vitro processing.

Here we report that the repressible *MET17* promoter produced the highest levels of hBD2 under initially repressing conditions, which was achieved in half the incubation time compared to the strong constitutive *PRB1* promoter in a yeast system modified for high-level production of biopharmaceutical proteins. Normally, the *PRB1* promoter is regulated by carbon and nitrogen sources as well as growth phase, which results in a transcriptional derepression of the *PRB1* promoter as the cells approach the stationary phase of growth [26]. However random mutagenesis of this proprietary yeast strain produced a constitutive *PRB1* promoter system, which allows expression of the target protein during exponential growth as well as during late exponential growth [27].

## Results

### High-level expression of human β-defensin-2 from the *MET17* promoter is induced by methionine depletion

To determine if the repressible *MET17* promoter was more efficient for hBD2 expression than the strong constitutive *PRB1* promoter, expression plasmids were produced harbouring the gene for hBD2 downstream of either the *MET17* or *PRB1* promoter. An expression plasmid comprising the *MET17* promoter upstream of the rHA gene was also made to compare the expression of a non-toxic protein to that of a toxic protein. A low productivity *S. cerevisiae* strain, DB1 and a high productivity *S.* *cerevisiae* strain, DYB7, were used for these studies (Table 1).

**Table 1:** Yeast strains used in this work

Strain	Known genotype	Reference
DB1	cir^0^, *MAT*a, *leu2*-*3, leu2*-*112*	[27]
DBY7	rHA-overproducing strain derived from DB1 by chemical mutagenesis. cir^0^,* MAT*a*, leu2*-*3, leu2*-*112, ubc4, ura3, yap3::URA3, lys2, hsp150::LYS2*	[39]

**Fig. 1:**
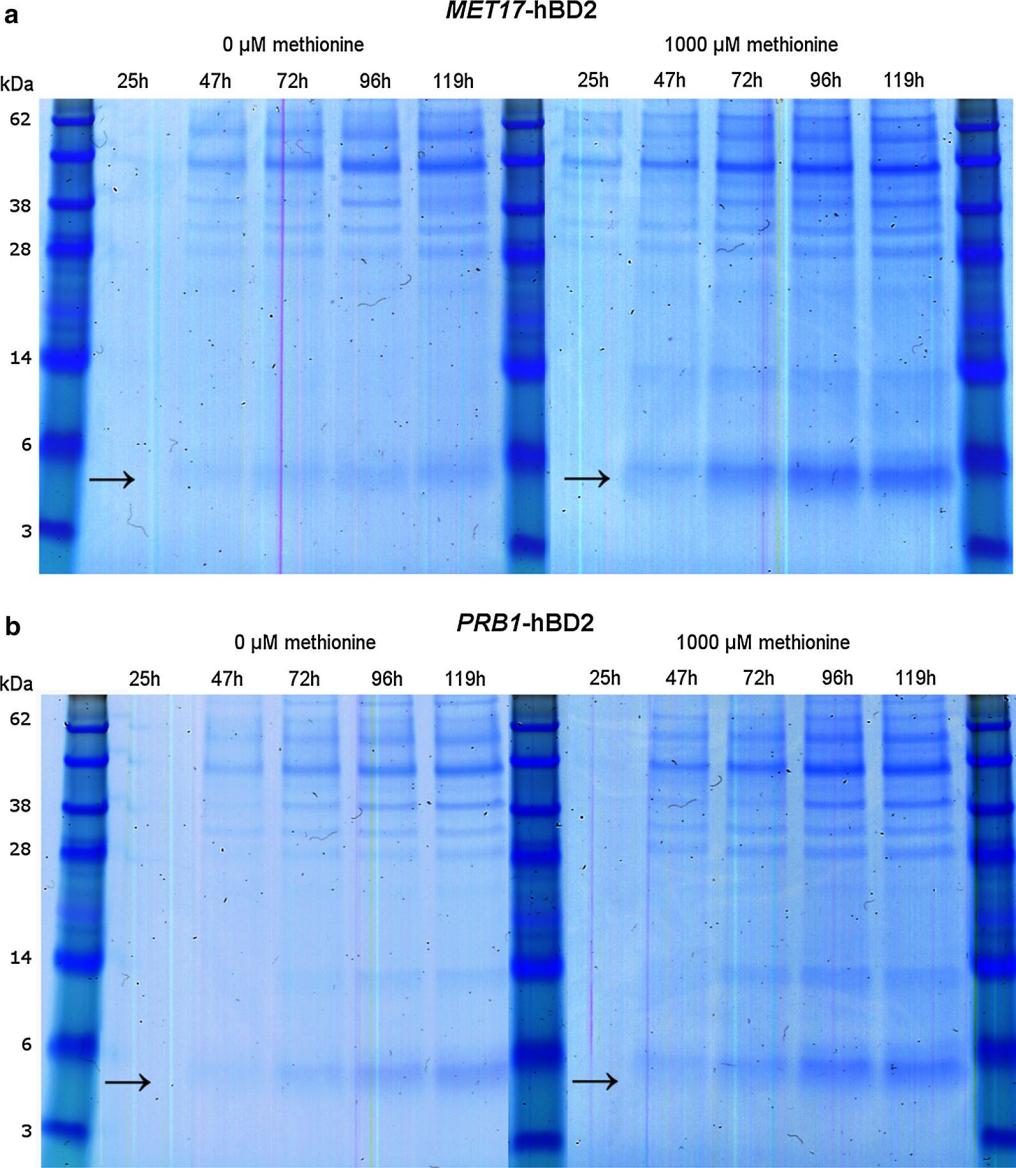
hBD2 secretion from yeast strain DYB7 using the *MET17* and *PRB1* promoters. SDS-PAGE analysis for hBD2 secretion from the *MET17* and PRB1 promoter with and without methionine applied to the growth media. 15 µL of BMMD SFC supernatant was analysed per lane by SDS-PAGE (NuPAGE^®^ 12% Bis–Tris, MES SDS running buffer, Invitrogen). 10 μL SeeBlue^®^ Plus2 Pre-stained protein standard (Invitrogen). Culture samples were taken approximate every 24 h during the 5-day incubation. hBD2 is marked with *arrows*. **a** Expression of hBD2 from the *MET17* promoter under non-repressing (0 µM methionine) and initially repressing conditions (1000 µM methionine). **b** Expression of hBD2 from the *PRB1* promoter without methionine (0 µM methionine) and with methionine in the media (1000 µM methionine)

**Fig. 2:**
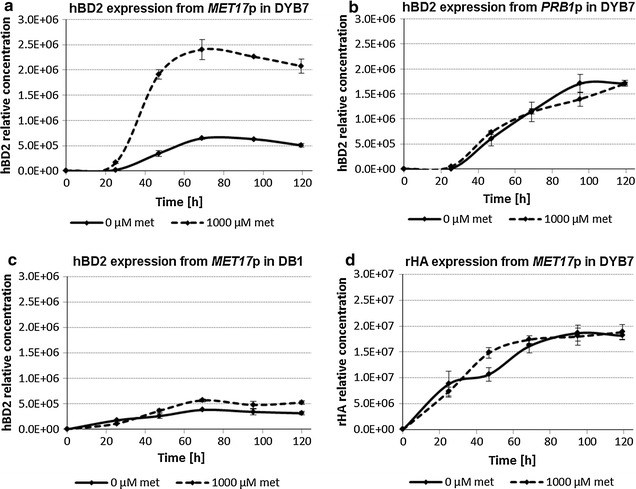
Relative hBD2 and rHA productivity determined by UPLC-MS analysis. hBD2 and rHA were expressed using either the *MET17* or the *PRB1* promoter on 2 µm-based expression plasmids. Plasmids were transformed into *S.* *cerevisiae* strain DYB7 or DB1. The transformed yeast were inoculated at OD_600_ = 0.15 into BMMD SFC without or with 1000 µM methionine and grown for 5 days, while culture samples were taken approximately every 24 h. *Error bars* indicate standard deviations (n = 3). **a** Expression of hBD2 from the *MET17* promoter (pDB3936:GR:pDB4351) under non–repressing (0 µM methionine) and initially repressing conditions (1000 µM methionine) in DYB7. **b** Expression of hBD2 from the *PRB1* promoter (pDB3936:GR:pDB4146) without methionine (0 µM) and with methionine in the media initially (1000 µM) in DYB7. **c** Expression of hBD2 from the *MET17* promoter (pDB3936:GR:pDB4351) under non-repressing (0 µM methionine) and initially repressing conditions (1000 µM methionine) in DB1. **d** Expression of rHA from the *MET17* promoter (pDB3936:GR:pDB4692) under non-repressing (0 µM methionine) and initially repressing conditions (1000 µM methionine) in DYB7

Shake flask cultures of yeast strains were grown in non-repressing (methionine-free medium) and repressing (1000 µM initial methionine concentration) conditions to control protein production. Analysis of the SFC supernatants of DYB7 [pDB3936:GR:pDB4351], expressing hBD2 from the *MET17* promoter, by SDS-PAGE (Fig. 1) and ultra-performance liquid chromatography mass spectrometry (UPLC-MS) (Fig. 2) revealed that the hBD2 expression was significantly improved by initial repression of the *MET17* promoter. Results for the *PRB1* promoter driven constructs confirmed that derepression only occurred with the *MET17* promoter constructs. The UPLC-MS data indicated that a higher hBD2 concentration was obtained in DYB7 using the *MET17* promoter repressed by 1000 µM methionine compared to the *PRB1* promoter in less than half the incubation time (Fig. 2a, b). The hBD2 yield using the *MET17* promoter was estimated to be approximately double that of the *PRB1* promoter after 70 h. This demonstrated that the expression of antimicrobial defensin hBD2 from the repressible *MET17* promoter was advantageous and resulted in a higher overall yield (g/L/h), an important factor for the economics of biopharmaceutical manufacturing.

In order to demonstrate the effect of strain optimisation from successive rounds of chemical mutagenesis and selection, production of hBD2 by the *MET17* promoter was also analysed in the original progenitor strain DB1 and compared to a mutated high-level production strain DYB7 [27–30]. In SFC, DYB7 showed an approximate four-fold increase in hBD2 production levels compared to the original progenitor strain DB1, under initially repressing conditions (Fig. 2a, c). Furthermore, the benefits of repressing hBD2 expression early in the growth phase were greater in the higher productivity strain DYB7.

Recombinant human albumin (rHA) expression from the *MET17* promoter was used as a control for expression of a non-toxic protein compared to hBD2 (Fig. 2d). Similar rHA protein concentrations were obtained after 120 h of expression regardless of whether the *MET17* promoter was repressed or not in DYB7. Therefore, this UPLC–MS data indicates that the *MET17* promoter is a valuable tool for the production of toxic proteins in this expression system.

### High hBD2 mRNA levels were obtained from a derepressed *MET17* promoter

**Fig. 3:**
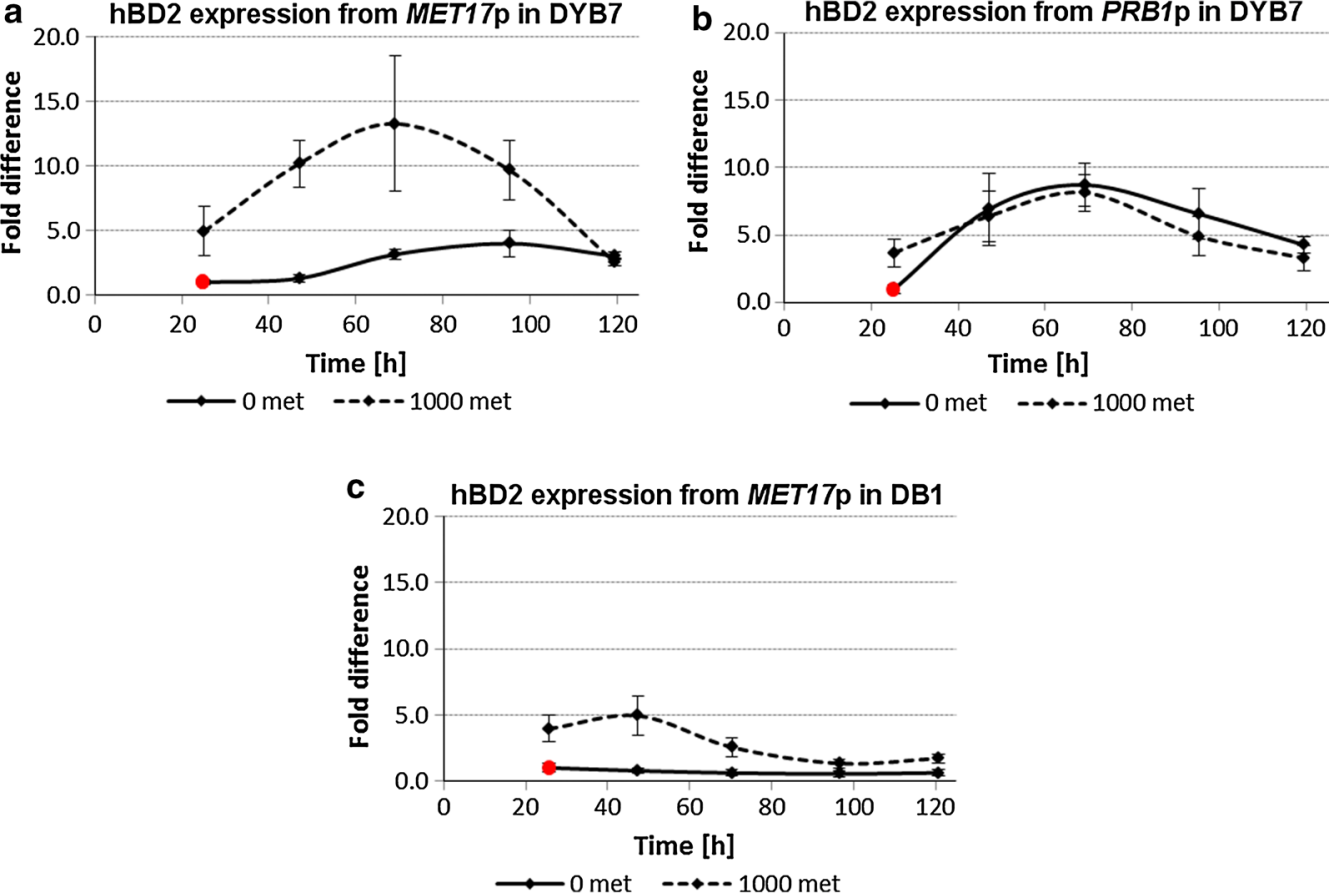
Transcript levels determined by real-time PCR. hBD2 was expressed from a 2 µm-based expression plasmid containing either the *MET17* or *PRB1* promoter upstream of the hBD2 gene. These plasmids were transformed into *S. cerevisiae* strains DYB7 or DB1. The transformed yeast were inoculated at OD_600_ = 0.15 into BMMD SFC without or with 1000 µM methionine and grown for 5 days, while cell pellet samples were taken approximately every 24 h and stored in RNAlater (Invitrogen) before RNA isolation and subsequently cDNA preparation. *Error bars* indicate coefficient of variation (n = 6). The fold difference is relative to the culture without methionine at 24 h in each strain (marked in *red*). **a** hBD2 mRNA produced from DYB7 with the *MET17* promoter under non-repressing (0 µM methionine) and repressing conditions (1000 µM methionine). **b** hBD2 mRNA produced from DYB7 with the *PRB1* promoter without methionine added to the media (0 µM) and with added methionine (1000 µM). **c** hBD2 mRNA produced from DB1 with the *MET17* promoter under non repressing (0 µM methionine) and repressing conditions (1000 µM methionine). *TAF10* was used as the endogenous control

Derepression of the *MET17* promoter was demonstrated in quantitative RNA studies from both *S.* *cerevisiae* DB1 and DYB7 by marked increases in the hBD2 mRNA levels during the exponential growth phase when methionine was initially present in the media (Fig. 3a, c). This increase in mRNA was likely to be caused by the derepression of the *MET17* promoter as it was induced by methionine depletion during cell growth. It was observed that the maximum hBD2 transcript levels from the *MET17* promoter occurred earlier for DB1 than the higher productivity strain DYB7, which can be attributed to growth rate differences between these strains (see Fig. 5a, c). Significantly lower transcript levels were seen in the cultures without methionine in the media, which indicated that a higher overall mRNA level was obtained from an initially repressed *MET17* promoter. The transcript levels from the *PRB1* promoter-driven construct confirmed that derepression was specific to the *MET17* promoter constructs, as the *PRB1* promoter was not affected by the methionine concentrations used in this study. Changes in the transcript levels throughout the expression study are attributed to changing environmental conditions during these batch cultures.

A verification of two endogenous controls was performed to demonstrate which one was the most suitable endogenous control for the type of assay performed (results not shown). *ACT1* was chosen due to its history of frequent use as an endogenous control in RT-PCR experiments [31, 32]. *TAF10* (TATA binding protein associated factor) was selected based on the research by Teste et al. which demonstrated that it is a suitable endogenous control because its expression was the most stable under the conditions tested. The transcript levels of these genes were analysed by a direct comparison of their cycle threshold (C_t_), assuming equal C_t_ for equal transcript level, since all RT-PCR reactions were performed with equal quantity of total RNA (cDNA). The C_t_ values for *TAF10* displayed a smaller variation and gradient than the C_t_ values for *ACT1*. Consequently, *TAF10* appeared to be a better endogenous control than *ACT1* for the experiments performed in this study. Based on these results *TAF10* was used as the endogenous control all RT-PCR experiments.

### Plasmid copy-number was affected by promoter activity

**Fig. 4:**
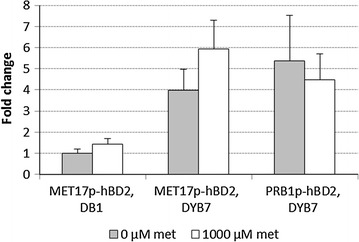
Relative plasmid gene copy-number determined from real-time PCRs. The transformed yeast were inoculated at OD_600_ = 0.15 into BMMD SFC without or with 1000 µM methionine and grown for 24 h. The cells were harvested and total DNA was isolated from 10 mL cultures. The plasmid names refer to the gap–repair plasmids containing the expression cassettes with the promoter and the expressed protein indicated below, all of which are present in the final pSAC35–based expression plasmid in yeast. Cultures were grown without and with (1000 µM) methionine in the initial media. *Error bars* represent the standard deviation of triplicate analysis of triplicate experimental cultures (n = 9). The fold change is relative to DB1 [pDB4351] without methionine. *FLP1* was used as the single-copy plasmid gene and *TAF10* was used as the genomic control

Significantly higher relative plasmid copy-numbers were obtained in the high productivity strain DYB7, compared to the original progenitor strain DB1, which is consistent with the *ubc4* mutation in DYB7 causing an increased plasmid copy–number (Fig. 4) [28]. The results obtained through copy-number determination using (RT-PCR) did not show a significant difference between cultures with and without methionine repression in any strain.

### Higher cell densities in hBD2 cultures with a repressed *MET17* promoter

**Fig. 5:**
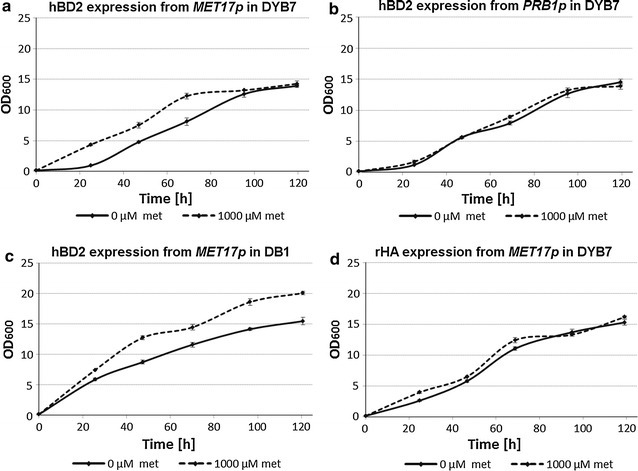
Growth of DYB7 and DB1 producing hBD2 or rHA. rHA and hBD2 were expressed using 2 µm expression plasmids containing either the *MET17* or *PRB1* promoter upstream of the coding regions. These plasmids were transformed into *S.* *cerevisiae* strain DB1 or DYB7. The transformed yeast were inoculated at OD_600_ = 0.15 into BMMD SFC with or without 1000 µM methionine and grown for 5 days, while OD_600_ measurements were done approximately every 24 h. *Error bars* indicate standard deviations (n = 3). **a** Growth of DYB7 [pDB3936:GR:pDB4351], which express hBD2 from the *MET17* promoter under non-repressing (0 µM methionine) and initially repressing conditions (1000 µM methionine). **b** Growth of DYB7 [pDB3936:GR:pDB4146], which express hBD2 from the *PRB1* promoter without methionine (0 µM methionine) and with methionine in the media initially (1000 µM methionine). **c** Growth of DB1 [pDB3936:GR:pDB4351], which express hBD2 from the *MET17* promoter under non-repressing (0 µM methionine) and initially repressing conditions (1000 µM methionine). **d** Growth of DYB7 [pDB3936:GR:pDB4692], which express rHA from the *MET17* promoter under non-repressing (0 µM methionine) and initially repressing conditions (1000 µM methionine)

In small scale cultures, a higher biomass was achieved earlier in the exponential growth phase in the repressed cultures of both DB1 and DYB7 with hBD2 expression from the *MET17* promoter (Fig. 5a, c). The repression effect was much more pronounced when the yeast cells were producing hBD2, compared to the albumin control (Fig. 5d), presumably because of the greater burden on host cell metabolism. The difference in growth between the cultures expressing rHA from the *MET17* promoter and the cultures expressing hBD2 from the same promoter are consistent with the antimicrobial activity of hBD2 inhibiting the cell growth. The *PRB1* promoter driven constructs (Fig. 5b) confirmed that the repression effect was specific to the *MET17* promoter in both low and high productivity *S. cerevisiae* strains. The antimicrobial effect of hBD2 was also observed on the growth curves of DYB7 when the *PRB1* promoter was used compared to the *MET17* promoter under initially repressing conditions, as a longer lag phase was observed (Fig. 5a, b). The growth inhibiting effect of hBD2 production was less marked for DB1 than for DYB7 (Fig. 5a, c), potentially due to the faster growth and lower productivity of DB1 compared to DYB7, suggesting that exceeding a critical hBD2 concentration for a given cell density may be required for cell growth to be inhibited.

Consequently, a higher biomass was obtained earlier using the *MET17* promoter initially repressed by methionine, because a separation of the hBD2 production phase and the yeast growth phase had been achieved.

## Discussion

For the cultures containing 1000 mM methionine in the initial medium it is interesting that this does not appear to have contributed to a significant increase in growth rate compared to the cultures lacking this additional nutrient (Fig. 5b, d). Neither does it appear to have had a significant effect on the production of either hBD2 or rHA (Fig. 2b, d), suggesting it has not alleviated a limitation to their secretion. It is however, possible that overall protein expression might have been affected and background staining of SDS-PAGE gels appears to be greater for cultures with 1000 mM methionine in the initial media (Fig. 1), which is otherwise a minimal medium with micronutrients supplemented, but devoid of amino acids. If so, this effect could add to the stimulatory effect of initial repression of hBD2 and further boost the production of the target protein.

It is interesting that the increase in hBD2 expression due to initial repression with methionine was so much greater for the higher productivity strain DYB7 compared to the progenitor strain DB1. This effect might be attributed to the greater toxicity caused by unregulated higher level hBD2 secretion, both intracellularly and also in the culture medium, where a threshold hBD2 concentration may be required for inhibitory effects on the host. While the hBD2 inhibition of *Candida* species is reported to vary considerably between strains in the range of 3.9 µg/mL to > 250 µg/mL [21], the inhibitory concentration for DB1 and DYB7 remains to be defined, and indeed may differ due to the mutagenesis perform on DYB7.

Due to the low biomass available at early time points in this study, the first transcript data were at 24 h, where hBD2 mRNA levels already appeared to be substantially higher when methionine was initially present in the culture media (Fig. 3a, c). Indeed, for both DYB7 and DB1 the hBD2 transcript levels are higher for the initially repressed cultures over the entire time course, suggesting that they had overshot the levels in the non-expressed cultures before 24 h. It is possible that by this time the increased biomass shown in Fig. 5a, c for the initially repressed cultures might alleviate the toxic effects on the culture during the next 24 h period when the majority of hBD2 was produced (Fig. 2a).

Although it is clear from Fig. 4 that DYB7 has a higher plasmid copy number than DB1, the differences between repressed and non-repressed cultures with hBD2 expressed from the *MET17* promoter are not significant in this study. Further analysis is therefore required to establish whether *MET17* promoter transcription impacts on the transcription of the *REP1, REP2, FLP1* and *D* genes, which might affect the plasmid copy-number through their roles controlling plasmid amplification and partitioning between mother and daughter cells [33, 34].

While accurate quantification of the mature hBD2 expression levels has not been performed in this study, the intensity of bands observed by SDS-PAGE suggests that high-levels of hBD2 production might be achieved during large scale fermentation using this well characterised yeast system. Due to the inherent scalability of this yeast system and its extensive industrial use for the production of human albumin and other recombinant proteins, expression levels of several hundred milligrams per litre of this 4.3 KDa protein might be expected, based on the equivalent molar expression levels of other proteins produced at large scale. Furthermore, the encouraging results described here, suggest that optimisation of methionine concentration during high-cell density fermentation could be used to maximise hBD2 yields when using the *MET17* promoter for large scale production. It would also be interesting to know at which time point the methionine is depleted and to perform metabolomics studies of the intracellular amino acid pools. Overall, the successful track–record of this yeast expression platform for the production of approved biopharmaceutical proteins and the economic benefits of secreting active soluble hBD2, without the need for expensive in vitro processing, make it an attractive candidate for further studies to be performed at large scale [35].

## Conclusions

Improved production of hBD2 was obtained in shake flask culture using the repressible *MET17* promoter under initially repressing conditions. Higher final concentrations of hBD2 were obtained using the repressible *MET17* promoter compared to the strong constitutive *PRB1* promoter in a *S. cerevisiae* strain developed for the high–level commercial production of recombinant proteins such as human albumin. Furthermore, the *MET17* promoter system gave a higher hBD2 concentration in less than half the time of the *PRB1* promoter system in this industrial yeast system. It was also shown that protein production could be controlled by the *MET17* promoter through addition of methionine to the media, which was advantageous for the production of hBD2 and may also be beneficial for other proteins that are deleterious to the host. Overall, these results showed great potential to using the *MET17* promoter for optimising production of difficult proteins from *S.* *cerevisiae,* which is likely to be applicable to large scale manufacturing of biopharmaceutical proteins.

## Methods

### Plasmids and yeast strains

Plasmid pDB4081 contains the *PRB1* promoter, a secretory leader sequence, the coding sequence for rHA and the *S. cerevisiae ADH1* transcription terminator. The DNA sequence of the *PRB1* promoter is −819 to −1 bps from the start codon of the *PRB1* gene on Chr. V 40046-41953 [GeneID:856649]. The DNA sequence for rHA is a human albumin sequence [GeneID:213] codon optimised for expression in *S.* *cerevisiae*. The 5′-end of the human albumin DNA was altered to replace the native secretory leader sequence with a modified version of the pre–pro HSA/*MFα1* fusion leader sequence [30] consisting of the amino acid sequence MKWVFIVSILFLFSSAYSRSLDKR (referred to as mHSA/*MFα1*-leader) [18]. Additionally, the 3′-end of the human albumin DNA was modified to introduce two TAA stop codons. pDB4692 was constructed by cloning the *MET17* promoter in place of the *PRB1* promoter in pDB4081. The DNA sequence of the *MET17* promoter is −388 to −1 bps from the start codon of the *MET17* gene on Chr. VII 732542–733876 [GeneID:851010]. The hBD2 DNA fragment was cloned between the *PRB1* promoter and the *ADH1* terminator in pDB4081 to replace the rHA gene and create pDB4146. The human β-defensin-2 sequence on Chr. 8 7752099–7754237 was codon optimised for expression in *S. cerevisiae* and an additional TAA stop codon was applied to the sequence. pDB4351 was constructed by cloning the *MET17* promoter into pDB4146 in place of the *PRB1* promoter. The *MET17* and *PRB1* promoters in pDB4146 and pDB4351 are linked to the *MFα1* (mating factor alpha) leader sequence (MRFPSIFTAVLFAASSALAAPVNTTTEDETAQIPAEAVIGYLDLEGDFDVAVLPFSNSTNNGLLFINTTIASIAAKEEGVSLD, [GeneID:296148471]) fused to the plectasin pro sequence plus a Kex2 protease cleavage site, with the sequence APQPVPEAYAVSDPEAHPDDFAGMDANQLQKR. These expression cassettes were cloned into a 2 µm plasmid, pDB3936 derived from of pSAC35 [36] and transformed into *S. cerevisiae* DB1 and DYB7 simultaneously for gap–repair recombination [37, 38]. In pDB3936, the *LEU2* gene of pSAC35 has been truncated by replacing *Kpn*I–*Not*I fragment with a synthetic linker, resulting in removal of the *Not*I site. Plasmids pDB4081, pDB4146, pDB4351 and pDB4692 contain the rHA or hBD2 expression constructs flanked by DNA for homologous recombination with pDB3936. Gap-repair transformants harbouring yeast expression disintegration plasmids were obtained by introducing purified *Acc*65I-*Bam*HI “gapped plasmid” DNA (pDB3936) and purified *Pvu*I-*Nsi*I “insert” DNA. Plasmids obtained through gap-repair were named on basis of the origin of the “gapped plasmid” (pDB3936) and the plasmid of the “insert” fragment (e.g. pDB4692) using the convention pDB3936:GR:pDB4692. The yeast strains and expression plasmids used in this study are listed in Tables 1 and 2, respectively.

**Table 2:** Description of plasmids used in this work

Plasmid	Description
pDB3936	pSAC35, a 2 μm-derived yeast episomal expression vector with a truncated *LEU2* [36]
pDB4081	Containing the expression cassette; *PRB1* promoter, the mHSA/*MFα1*-leader leader, the coding sequence of rHA and the *S. cerevisiae ADH1* transcription terminator
pDB4146	Containing the expression cassette; *PRB1* promoter, the *MFα1/*plectasin leader, the coding sequence of hBD2 and the *S. cerevisiae ADH1* transcription terminator
pDB4351	Containing the expression cassette; *MET17* promoter, the *MFα1/*plectasin leader, the coding sequence of hBD2 and the *S. cerevisiae ADH1* transcription terminator
pDB4692	Containing the expression cassette; *MET17* promoter, the mHSA/*MFα1*-leader leader, the coding sequence of rHA and the *S. cerevisiae ADH1* transcription terminator

### Culture conditions

Shake flask cultures with 10 mL BMMD (buffered minimal media dextrose [28]) were grown in 50 mL conical shake flasks to an OD_600_ of 1.5–2.5 at 30 °C, 200 rpm, 25 mm orbit. 670 µM methionine was used in the hBD2 expressing pre-cultures to repress hBD2 expression. The amount of this pre-culture required to inoculate the main culture at OD_600_ of 0.15 was calculated, pelleted by centrifugation and washed with BMMD medium to eliminate any methionine used in the pre-culture. This washed cell pellet was then resuspended in the appropriate volume of BMMD with the correct concentration of methionine and grown for 5 days at 30 °C, 200 rpm in 50 mL conical shake flasks.

### SDS–polyacrylamide gel electrophoresis (PAGE)

Culture supernatant was run on NuPAGE^®^ 12% Bis–Tris gels (Invitrogen) in MES SDS running buffer at 200 V for 35 min. The molecular weight standard used was SeeBlue^®^ Plus2 Pre-stained protein standard (Invitrogen). Proteins were stained using InstantBlue (Expedeon).

### Ultra-performance liquid chromatography mass spectrometry (UPLC-MS)

Chromatographic separation of culture supernatants was carried out on an ACQUITY UPLC system (Waters) using an ACQUITY UPLC BEH 300 Å C-18 column (2.1 mm id ×100 mm, particle size 1.7 μm) at 45 °C with a flow rate of 0.250 mL/min. The mobile phase consisted of 0.1% formic acid in Milli-Q H_2_0 (solvent A) and 0.1% formic acid in acetonitrile (solvent B). The column was eluted using a gradient from 5% B to 45% B over 4 min and 30 s. The injection volume was 10 μL. UPLC column eluate was analysed by online electrospray ionization-time of flight mass spectrometry using a calibrated micrOTOF II (Bruker) mass spectrometer in positive ion mode. Data were analysed for relative quantitation using DataAnalysis 4.0 (Bruker) software by extracting the ion chromatogram (EIC) for the 722 mass-to-charge (*m/z*) analyte. Charge state deconvolution, to confirm the intact mass of the analyte, was performed using the maximum entropy method.

### Total DNA extraction

Strains were grown at 30 °C with shaking (200 rpm, 25 mm orbit) for approximately 24 h in 10 mL BMMD medium with and without the appropriate methionine concentration at 30 °C to an OD_600_ in the mid-log phase. Cells were harvested by centrifugation at 5000*g* for 5 min and stored at −80 °C. Total DNA was isolated using a Wizard^®^ Genomic DNA isolation kit (Promega). DNA quantity was determined by A_260_ measurements and purity by A_260_/A_280_ ratio measurements using a NanoDrop 1000 UV–Vis spectrophotometer (Thermo Scientific).

### Total RNA extraction and cDNA synthesis

Every 24 h 500 µL culture samples were taken and cells harvested by centrifugation at 13,000*g* for 2 min. The pellets were resuspended in 300 µL RNAlater (Qiagen) and stored at 4 °C for immediate stabilisation and protection of the RNA. Total RNA was extracted using the RNeasy Mini kit (Qiagen) with yeast spheroplasts prepared by lyticase (Sigma) digestion. To eliminate genomic DNA contamination, an additional DNase treatment was performed according to the RNeasy kit instruction with the RNase-free DNase set (Qiagen). Quantity of the extracted RNA was determined by A_260_ measurements and purity by A_260_/A_280_ ratio measurements using a NanoDrop 1000 UV–Vis spectrophotometer (Thermo Scientific). 100 ng DNase digested total RNA was used in a 20 μL Superscript III (Invitrogen) reverse transcriptase reaction mixture according to the manufacturer’s instructions.

### Quantitative real time polymerase chain reaction (RT-PCR)

TaqMan^®^ primer/probe sequences were designed using Primer Express software (Applied Biosystems). Reactions were set up in total volumes of 25 μL comprising 12.5 μL of 2× TaqMan^®^ Gene Expression master mix (Applied Biosystems), 250 nM probe, 1 μM primers and 5 μL of cDNA or DNA template (diluted 100-fold). The absence of genomic DNA contamination in RNA samples were checked by RT-PCR before cDNA synthesis using RNA as a template in RT-PCR (minus reverse transcriptase control). Blank samples (no template control) were incorporated in each assay. Reactions were carried out in triplicates and as singleplex reactions using an Applied Biosystems 7500 system with the thermocycling program consisting of one hold at 50 °C for 2 min, another hold at 95 °C for 10 min, followed by 40 cycles of 15 s at 95 °C and 1 min at 60 °C. Transcript data were analysed using the relative standard curve method using *TAF10* as endogenous control and gene copy-numbers were determined using *FLP1* as the single-copy reference gene.

## Abbreviations

BMMD: basal minimal media dextrose
Ct: cycle threshold
EIC: extracted-ion chromatogram
hBD2: human β-defensin-2
HSA: human serum albumin
MES: 2-(N-morpholino) ethanesulfonic acid
rHA: recombinant human albumin
RT-PCR: reverse transcription polymerase chain reaction
SDS-PAGE: sodium dodecyl sulfate polyacrylamide gel electrophoresis
SFC: shake flask culture
UPLC-MS: ultra-performance liquid chromatography mass spectrometry
